# Public Awareness of and Action towards Heart Attack Symptoms: An Exploratory Study

**DOI:** 10.3390/ijerph17238982

**Published:** 2020-12-02

**Authors:** Abdullah Abdulmajid Abdo Ahmed, Abdulkareem Mohammed Al-Shami, Shazia Jamshed, Abdul Rahman Fata Nahas, Mohamed Izham Mohamed Ibrahim

**Affiliations:** 1 Department of Pharmacy Practice, International Islamic University Malaysia, Kuantan 25200, Pahang, Malaysia; abdullahalbohari@gmail.com (A.A.A.A.); Dhamarali2002@yahoo.com (A.M.A.-S.); pharmacist1992@live.com (S.J.); abd_mfn@iium.edu.my (A.R.F.N.); 2 Qualitative Research-Methodological Application in Health Sciences Research Group, Kulliyyah of Pharmacy, International Islamic University Malaysia, Kuatan 25200, Pahang, Malaysia; 3 Clinical Pharmacy and Practice Department, College of Pharmacy, QU Health, Qatar University, P.O. Box 2713, Doha, Qatar

**Keywords:** heart attack, awareness, symptoms, action towards, appropriate action, public health

## Abstract

Background: Cardiovascular disease (CVD) is still the leading cause of mortality worldwide, and Malaysia is not an exception in this regard. The current research is an attempt to explore symptom awareness of and necessary actions in response to heart attack (HA) among lay public. Methods: This is a cross-sectional study design, and a survey was conducted from May to July 2018 among general public in Kuantan, Pahang state, Malaysia. Results: A total of 393 respondents recruited. Slightly more than one-fourth of the respondents (26.35%) were aware of HA symptoms like pain and/or discomfort in the jaw, neck, or back, while 71.65% showed awareness only of chest pain or discomfort as symptoms. Only 35.6% reported to call an ambulance if they experience someone suffering from HA symptoms, while 82% recognized ≥1 symptom, and only 11.5% recognized all five HA symptoms. Very few respondents, i.e., 1.3% reported awareness about correct recognition of all five HA symptoms. Respondents who had diabetes and hypercholesteremia were more likely to recognize all five HA symptoms. For those who had excellent awareness of all five HA symptoms, the odds ratio (OR) were significantly higher among single respondents (OR 0.023; 95% CI 0.001–0.594), Malay (OR 0.376; 95% CI 0.193–0.733), and those who received information associated with HA (OR 7.540; 95% CI 2.037–27.914). However, those who were aware that HA requires quick treatment had significantly low odds ratio (OR 0.176; 95% CI 0.044–0.710). Conclusions: The awareness of and action towards the signs and symptoms of HA among the public were poor.

## 1. Introduction

Cardiovascular disease (CVD) is still the leading cause of mortality worldwide, and Malaysia is not an exception in this regard. According to the recent statistics, 43.8% of people died due to CVD in 2018 [1]. Every 40 s, about one American suffers from heart attack (HA), a common terminology for myocardial infarction (MI). In Malaysia, coronary heart disease (CHD) is the leading cause of mortality [2]. According to the World Health Organization (WHO), CHD accounted for 23.10% of all deaths in Malaysia in 2014. CHD is also known as ischemic heart disease (IHD), and according to the government of Malaysia, IHD is the main cause of death; approximately 13.2% of people died in 2016 [3]. Previous studies have illustrated that more than 50% of HA deaths occur within 1 h of symptoms prior to patient admission to a medical center [4,5].

The onset of HA and taking proper immediate action can have a significant impact on morbidity and mortality [6] Among patients suffering from HA, a longer prehospital delay to obtain emergency medications can negatively affect the patient prognosis [7,8,9]. Annually, thousands of people die or suffer permanently because no appropriate action is taken to respond to HA symptoms. HA typically appears as chest pain or discomfort that may spread to the arms, neck, jaw, and back and be accompanied by difficulty in breathing and sweating [10]. Furthermore, in some diabetic or elderly patients, HA can sometimes occur silently and with atypical symptoms, such as abdominal pain [11]. Importantly, there can be gender differences in the symptoms [12]. In most conditions, the patient’s description of any symptoms is considered together with electrocardiogram results and cardiac-specific enzyme assays where the healthcare professional can only decide on an intervention or treatment. HA is also a time-dependent illness that leads to better results when patients receive medical care promptly after the onset of symptoms. Early admission to a hospital is vital to reduce HA complications as it leads to the improved delivery of emergency treatments. For instance, thrombolytics has optimal effects when used within 24 h; however, their administration during the first 60 min has shown to have better outcomes [13]. Pre-hospital delay is known as patients delaying calls for an ambulance or health care assistance and delaying transportation to a hospital [14]. This delay may occur due to a lack of awareness of HA symptoms, which increases death rates. Other patients delay perhaps due to denial, fear, and unwarranted trust in self-administration, and several patients may fear embarrassment if they report to the hospital needlessly.

## 2. Material and Methods

### 2.1. Study Aim

The current research is an attempt to explore the awareness of symptoms and actions towards HA among lay public in Kuantan, Pahang, Malaysia.

### 2.2. Design and Respondents

This is a cross-sectional study design in which 393 respondents were recruited by means of convenience sampling in Kuantan, a city located on the eastern coast of Malaysia. The participants were approached at different local shopping centers and malls, mosques, churches, and eateries. The participants were selected conveniently based on the accessibility and intention to participate in the study. The participants were apprised of the information about the study and asked for their consent to participate. Those who consented to participate signed an informed consent form. The participant had full liberty to leave the study at any point of time. An interviewer-administered questionnaire was used to collect the information from the participants. Respondents were in the age-range of 18–64 years old and were guaranteed that all their information would be kept confidential and used only for the research purpose. The study received approval from the International Islamic University Research Ethics Committee (IREC 2018-132).

### 2.3. Research Instruments

A pre-validated structured questionnaire was used which was developed by Ahmed et al., 2019 [15]. The first part of the questionnaire included demographic characteristics (age, gender, marital status, race, level of education, monthly income, and employment status). A second part included history of HA, family experience of HA, work environment, and sources of information about past history of hypertension, diabetes, coronary artery disease, or hyperlipidemia); additionally, data on history (diagnosis) of a HA of the respondents, their immediate family members, or their acquaintances were also collected. Moreover, questionnaires were included to collect data on social habits, such as smoking, alcohol consumption, and drug utilization. A total of 11 items were used to assess the level of awareness of the symptoms of and appropriate action in response to a HA. With regard to the awareness of the symptoms of and appropriate action in response to a HA, the respondents were asked about the following symptoms of HA: sudden pain or discomfort in the jaw, neck, or back; dizziness or weakness; sudden pain or discomfort in the chest; pain or discomfort in the arms or shoulders; and difficulty breathing. Those who answered “yes” to the questions were identified as having awareness of symptoms. Respondents who answered “no” or “don’t know” were classified as not having awareness of the symptoms. However, the trap question (sudden vision trouble) was included to assess and account for the possibility that the respondent would answer “yes” to all symptoms. Additionally, to assess the awareness of appropriate actions in response to symptoms, the respondents were asked, “If you see someone suffering from a heart attack, what do you think you should do first?” Respondents then selected from the list of actions that included taking patients to the hospital or clinic, telling him or her to call the doctor, calling an ambulance, calling the police, contacting their family, or other actions.

### 2.4. Data Collection

Potential respondents were approached through visiting public areas as mentioned above, and a detailed explanation of the study was provided. Face-to-face survey then was conducted, for data collection, by three trained personnel who speak Malay and English. Data collection was performed over two months, from 7 May 2018 to 10 July 2018.

### 2.5. Statistical Analysis

SPSS software version 23 (IBM Corp. Released 2015. IBM SPSS Statistics for Windows, Version 23.0. Armonk, NY, USA: IBM Corp.) was used to run statistical analyses. Description of respondents’ sociodemographic characteristics were performed. Mann–Whitney and Kruskal–Wallis tests were used for measuring differences of ordinal variables. Chi-square tests were conducted to differentiate individuals’ awareness of symptoms and appropriate action based on sociodemographic characteristics, including gender, age, level of education, and monthly income, in addition to risk factors such as family history of heart disease, hypertension, diabetes, hypercholesteremia, and stroke. All tests were carried out at alpha level of 0.05. Multivariate logistic regression analysis was performed to obtain the odds ratios (ORs) and 95% confidence intervals (CIs) for the variables affecting awareness of five HA symptoms and appropriate action. The regression was used to determine the relationship between each of the factors affecting awareness and all five HASs. The independent variables that were tested by the chi-square test include the sociodemographic of respondents, hypertension, dyslipidemia, family history of HA, and those who had received information about HA through announcements or the internet as well as those who were aware that HA requires immediate care. Variables with a significant *p*-value and with a *p*-value of less than 0.25 were included in this logistic regression model [16,17]. Therefore, the model included the following variables: marital status; race; education; monthly income; diagnoses of hypertension, diabetes, and dyslipidemia; those who have received information about HA through announcements or the internet; those who had a family history of HA; and those who were aware that HA requires prompt treatment.

## 3. Results

### 3.1. Demographic Characteristics

A total sample of 393 individuals included 204 (51.9%) men and 189 (48.1%) women residing in Kuantan. Table 1 shows that the largest group of respondents (31%, n = 122) were 18–25 years old. In addition, 53.7% of respondents were married, and 52.2% of them had a high school education. Moreover, about half of the respondents (50.9%) were employed, and only 16.5% of the respondents were students. We divided race into four groups; the majority of respondents were Malay (54.2%). Most of the respondents (68.4%) had monthly incomes of less than RM 2000 (≈USD 495). Additionally, approximately 17% of individuals were diagnosed with hypertension, 12% with diabetes, and 15% with hypercholesteremia.

### 3.2. Awareness of Each Symptom of a Heart Attack and Appropriate Action (Calling an Ambulance)

Awareness of the early signs and symptoms of a heart attack and appropriate action at the time of its occurrence (for instance, calling an ambulance) in relation to demographic characteristics and those who are at high risk are shown in Table 2. The majority of respondents (71.6%) recognized sudden pain or discomfort in the chest as heart attack symptoms (HAS). This was followed by shortness of breath (68.1%), weakness or dizziness (58.1%), and sudden pain or discomfort in the arms or shoulders (28.2%). On the other hand, the lowest percentage of the respondents (26.35%) recognized sudden pain or discomfort in the jaw, neck, and back as HAS. Furthermore, only 35.9% of the respondents were aware that the trap question (sudden disturbance of vision in one or both eyes) was not HAS. However, 11.5% of the respondents answered yes to all 5 HAS and trap questions, while 10.4% answered “no” to all HASs and trap questions. This implies that 22.9% of our sample were not aware of HAS. In addition, 35.6% of respondents identified calling an ambulance as a suitable action when someone is experiencing HASs. Overall, the Mann–Whitney U test showed that females had a greater awareness of difficulty breathing than males (U = 35,347, *p* = 0.038). In addition, Kruskal–Wallis (KW) test showed that respondents who were single had better awareness of sudden pain or discomfort in the jaw, neck, or back than others (*x*^2^ = 15.412, *p* = 0.001) and sudden pain or discomfort in the arms or shoulders (*x*^2^ = 9.566, *p* = 0.023). KW test also demonstrated that Malay respondents reported greater awareness of sudden pain or discomfort in the jaw, neck, or back compared to other races (*x*^2^ = 7.109, *p* = 0.029). However, Chinese respondents showed better awareness of weakness or dizziness and difficulty breathing than did individuals of other races (*x*^2^ = 8.746, *p* = 0.013; *x*^2^ = 15.378, *p* = 0.000, respectively), while Indians demonstrated higher awareness of pain or discomfort in the chest than did individuals of other races (*x*^2^ = 17.084, *p* = 0.000).

Furthermore, KW test revealed that respondents with lower levels of education (lower school and high school) were more likely to identify chest pain or discomfort than were those with higher levels of education (*x*^2^ = 13.597, *p* = 0.034). In addition, those with less income demonstrated better awareness of sudden pain or discomfort in the jaw, neck, or back (*x*^2^ = 8.003, *p* = 0.046). Additionally, there were no statistically significant differences by employment status (*p* = 0.627), age (*p* = 0.116), hypertension (*p* = 0.194), dyslipidemia (*p* = 0.064), and HA signs and symptoms. Furthermore, Mann–Whitney U test demonstrated that diabetic respondents established greater awareness of sudden pain or discomfort in the chest than others without diabetes (U = 7445, *p* = 0.054). Respondents without diabetes had more awareness of sudden pain or discomfort in the jaw, neck, or back (U = 7245, *p* = 0.020). Regarding the family history of heart disease (heart attack) among families; respondents without a family history of heart disease (heart attack) among relatives, acquaintances, or close neighbors were significantly more aware than those with relatives who had experienced a past HA with sudden pain or discomfort in the jaw, neck, or back (U = 15,900, *p* = 0.000); weakness or dizziness (U = 15,330, *p* = 0.000); sudden pain or discomfort in the chest (U = 16,417, *p* = 0.003); and sudden shortness of breath (U = 16,849, *p* = 0.015). Furthermore, Mann–Whitney U test demonstrated that those who had heard about heart attacks were more likely to be aware of weakness or dizziness (U = 7646, *p* = 0.001), pain or discomfort in the chest (U = 7458, *p* = 0.000), and difficulty breathing (U = 7478, *p* = 0.000) than were those who had not heard about heart attacks. Moreover, the Mann–Whitney U test reported that those who received information related to HA through public service announcements and the internet had less awareness than others who did not receive information about chest pain (U = 13,236, *p* = 0.028); weakness or dizziness (U = 13,018, *p* = 0.024); pain in the jaw, neck, or back (U = 11,937, *p* = 0.000); and pain in the arms or shoulders (U = 11,969, *p* = 0.000). However, respondents who received any information about heart attack through public services demonstrated greater awareness in calling an ambulance than did others who did not (U = 13,302, *p* = 0.047) (Table 2).

### 3.3. Awareness of Heart Attack Symptoms Using the Number of Symptoms

Majority of respondents (81.9%) recognized at least one HAS, whereas only 11.5% indicated all five HASs (Table 3). Only 1.3% of the respondents had excellent awareness and recognized all five HAS with the response “yes” and the trap question with the response “no” and responded “yes” to calling an ambulance. Few respondents (2.8%) recognized all five HASs and trap question. Furthermore, 5.6% of the respondents recognized all five HAS and an appropriate action (calling an ambulance). However, KW test demonstrated that respondents aged 36-45 years old were more likely to be aware of HASs than were respondents in other age groups (*x*^2^ = 9.965, *p* = 0.041). The test also showed that widowed respondents showed higher awareness of all five HAS (*x*^2^ = 7.504, *p* = 0.05) than did others in terms of marital status. In addition, Malay individuals showed higher awareness of one HAS (*x*^2^ = 16.743, *p* = 0.000), two HAS (*x*^2^ = 11.180, *p* = 0.004), and three HAS (*x*^2^ = 5.275, *p* = 0.022). Chinese respondents showed greater awareness on the five HASs (*x*^2^ = 7.860, *p* = 0.020) than did other respondents. Respondents who were postgraduate students showed more awareness of all five HASs than did respondents with other levels of education (*x*^2^ = 12.873, *p* = 0.045). Moreover, those with monthly income between RM 4000 and 5999 showed better awareness on all five HASs and excellent awareness than did those of other income levels (*x*^2^ = 8.493, *p* = 0.037 and *x*^2^ = 13.241, *p* = 0.004, respectively). However, there were no significant differences between those who had hypertension and those without hypertension on the five HAS and excellent awareness (U = 10,267, *p* = 0.161 and U = 10,892, *p* = 0.884, respectively). Nevertheless, the Mann–Whitney U test demonstrated that diabetic respondents were more likely to be aware of the five HAS than others without diabetes (U = 7538, *p* = 0.012). However, there were no significant differences between diabetic respondents and nondiabetic respondents on excellent awareness (U = 8503, *p* = 0.624). Moreover, individuals who have been diagnosed with hypercholesteremia demonstrated greater awareness of the five HAS (U = 8944, *p* = 0.009), appropriate action (U = 9028, *p* = 0.001) and excellent awareness (U = 9689, *p* = 0.006) than did others without hypercholesteremia. Additionally, respondents who received information related to HA through public service announcements or the internet were more likely to be aware of all five HAS than others were (U = 13,207, *p* = 0.002 and U = 13,998, *p* = 0.018, respectively). Furthermore, those who were not aware that HA requires urgent treatment had a greater awareness of the five HASs than did others who were aware (*x*^2^ = 7.098, df = 2, *p* = 0.029), as shown in Table 3.

### 3.4. Assessment of Factors Affecting the Awareness of the Five Heart Attack Symptoms

Multivariable logistic regression showed that four variables significantly affected the excellent of HAS that awareness of all five HAS. Individuals who were single were 0.458 times more likely (estimated around 98% less likely than the compared group) to identify all five HAS than were widows (OR = 0.458, 95% CI = 0.219–0.962, *p* = 0.039). Furthermore, Malay respondents were 0.381 times more likely (estimated around 62% less likely than the compared group) to recognize all five HAS than were non-Malay respondents (OR = 0.381, 95% CI 0.196–0.741, *p* = 0.004). Similarly, those with a family history of heart disease (heart attack) among their relatives and neighbors were 4.022 times more likely (estimated around 4 times more likely than the compared group) to identify five HAS than were those without a family history of heart disease (heart attack) (OR = 4.022, 95% CI = 1.126–14.363, *p* = 0.03). In addition, respondents who received information about HA through public service announcements and/or the internet were 6.095 times more likely (estimated around 6 times more likely than the compared group) to know the five HAS than were those who did not receive any information about HA (OR = 6.095, 95% CI 1.787–20.792, *p* = 0.004). On the other hand, there is no statistically significant differences between participants who aware that HA requires urgent medication and others who were unaware that, as well as between individuals with risk factors for HA, such as hypertension, diabetes, and hypercholesteremia, and those without risk factors for HA (Table 4).

## 4. Discussion

The delay in reporting time from the appearance of HA symptoms until the presentation at a medical care center is globally seen, and this is particularly related to patient’s ability to identify the signs and symptoms of HA [18]. According to Moser et al. (2006), a few important barriers that increase the rate of mortality and morbidity include delay in transportation to the hospital, delay in intervention after arrival to the hospital, and long time taken in seeking medical care [6]. The current research was conducted among the lay public in Kuantan, Pahang, Malaysia to assess their awareness of, and action taken in response to, the signs and symptoms of a HA. It is imperative to understand HA symptoms for seeking treatment and reducing morbidity and mortality. The awareness of the respondents regarding the signs of HA was unconvincing. Similarly, the awareness of the appropriate action to call an ambulance (through 999) and the awareness of five HA symptoms were very low. Additionally, only a few respondents had greater awareness of HASs. The findings of this research are in accordance with a South Korean study wherein only 10.9% of the study respondents reportedly knew all five symptoms, while 3.1% had excellent knowledge [19,20].

The present research reported insufficient awareness of typical HA symptoms, such as chest pain, dyspnea, and weakness or dizziness. Similarly, the awareness of atypical symptoms, such as sudden pain or discomfort in the jaw, neck, or back and sudden pain or discomfort in the arm or shoulder, was low. These results are consistent with those of other studies [21,22]. A lack of awareness of atypical HA symptoms can lead to prehospital delay and increase morbidity and mortality rates. As previously mentioned, the findings of the National Registry of Heart Attack showed that one-third of patients arrive at the hospital without chest pain [23].

In the current study, chest pain was the most common symptom of HA identified by the respondents. This finding is consistent with those of other studies conducted in Kuwait, South Korea, the US, Poland, the Emirates, Greece, and Jordan [20,24,25,26,27,28,29]. In contrast, a study performed in Nepal reported that chest pain was the second-most important sign for HASs identification. Although the authors added that nearly half of the respondents were illiterate [30], the respondents had at least a high school education.

Sudden pain or discomfort in the jaw, neck, or back was the least-recognized HAS among the current respondents, and this is consistent with those of a study performed in Poland that indicated that the most-recognized HAS among respondents was chest pain, while the least-recognized HAS was pain or discomfort in the jaw, neck, or back [27]. Another study conducted in the US reported that half of the respondents identified pain or discomfort in the neck or back and jaw as HAS, while 92% of the respondents identified chest pain or discomfort [19]. Furthermore, a study carried out in South Korea demonstrated that the most-recognized HAS reported by the respondents was chest pain, while the least-recognized HAS was pain in arms or shoulders [20]. However, a direct comparison is difficult due to differences in some of the opinions offered or in the questions in past studies. Nevertheless, in the present study, the awareness of suitable action towards HAS was low (35.6%) compared to studies in Poland (87.4%; [27]), the US (86.8%; [19]), and South Korea (67%; [20]). In previous studies, especially in Poland, research on the awareness of suitable action towards HASs was performed among respondents who had experience and who were educated by their physicians [27]. Furthermore, the current research showed a low percentage of respondents who identified chest pain as HAS. This plays a pivotal role in the delay in seeking treatment at the medical health care center.

Although majority of the sample recognized chest pain or discomfort as the first HAS, the literature indicates that 33% of patients with a confirmed diagnosis of HA did not present chest pain [23]. The lack of chest pain or discomfort is a potent predictor of skipped beat diagnosis and delayed therapy.

Furthermore, in this study, the second-most common HAS was dyspnea, and this is similar to the findings of South Korean research in which 70.2% of the respondents reportedly having shortness of breath [20]; this statistic marked 72% in Poland [27] and 83.4% in the US [19]. Nevertheless, sudden pain or discomfort in the arm or shoulder was also indicative of all five heart attack symptoms. This is in line with the findings of other studies in Poland (27.5%; [27]) and Koura, Lebanon (32.9%; [20]). In contrast, research performed in the US reported that a very high percentage (85.6%) of the respondents recognized pain in the arms or shoulders [19,25]. The high level of awareness for each individual HAS and appropriate action was attributed to the level of education and ethnicity in the US compared with the present study in Kuantan. In our study, most of the respondents were among the lay public with a high school level of education [31].

Additionally, approximately half of the respondents in our study identified weakness and light-headedness or faintness as HAS. The results are similar to those of other studies in Korea [20], Nepal [30], and the US [19]. In contrast, a study conducted in Greece showed that faintness was considered by the lowest percentage of respondents among all five heart attack symptoms [26]. The majority of the respondents had low education and income compared to those in the current study in Kuantan, where the majority of the respondents had a high school education and commensurate income.

Regarding the suitable actions for someone suffering from HASs, less than half of respondents in the current study were aware of the appropriate action, which is calling an ambulance (999). Studies in different countries, however, reported otherwise. For example, in Poland [27], South Korea [20], and the US [19], the percentages of respondents who indicated calling an ambulance as an appropriate action were 87.4%, 67%, and 86%, respectively. As reported earlier, the awareness of HASs was low in this study compared to in previous studies [19,27] that reported high awareness of each individual HAS. The low awareness of HASs in the current study might be responsible for the low recognition of the appropriate actions to be taken. Thus, the outcomes of this study were not consistent with the recommendations of the American Heart Association with regard to the protective measures to be taken when someone is suffering from HASs [32]. Nevertheless, it is necessary to know the appropriate actions in the case of HA, but it is not adequate to avoid prehospital delay. People who do not understand or lack awareness of the symptoms of HA may respond more slowly in seeking medical care [22].

Regarding the association between sociodemographic characteristics and suitable action in response to HASs, our research did not show any association between the recognition of suitable action and age, level of education, income, and family history of HA among those who were at risk of HA. Though these outcomes are consistent with those of a study performed in Poland [27], the results are not in line with the findings of studies in the US [19] and South Korea [20]. In addition, the current study did not identify any relationship between appropriate action and sociodemographic factors. This outcome is consistent with those of a study conducted in Thailand that reported no association between sociodemographic characteristics and appropriate action [20].

Regarding the number of HASs, the current study found that the majority of the respondents identified at least one symptom, while less than a quarter did not know any HASs. Consistent with the current research, studies in Singapore reported that 85.1% of the respondents recognized one HAS [33], while New England studies reported recognition among 87% of respondents [34], and South Korea studies reported recognition among 88.7% of respondents [20]. In contrast, studies performed in Poland and the US showed that almost all the respondents recognized one HAS [19,27]. Another study in the US reported that 92% of the respondents recognized at least one HAS, and 31% identified all five HAS [5].

Furthermore, the current research demonstrated that 11.5% of the respondents identified all HASs, while 5.6% knew all HASs and the appropriate action to call an ambulance, and 1.3% had a high awareness of HASs. These findings are consistent with those of a study performed in Korea, Canada, and Poland [20,27,35]. In contrast, a study in the US reported that 32% of all respondents identified all HASs [19]. Additionally, the current research found a relationship between awareness of all five HASs and race, high level of education, monthly income, and those who are at a high risk of HA. The results are in agreement with other studies in the US, Poland, Korea, and Canada [19,20,25,27,35].

Regarding the awareness of all five HASs, a relationship was identified between the awareness of the need for urgent therapy and race, education, income, diabetes, family history of HA among relatives, and those who received information through social media. Our findings are similar to those of a study in Korea [20]. Furthermore, the results of the present research demonstrated an association between in-depth knowledge of all HASs and those who are at high risk of CVD. This finding is consistent with those of a study performed in the US that reported that respondents with a high risk of CVD were more knowledgeable than others without risk factors [25]. In contrast, the findings in this study are not congruent with those of a study performed in Korea that reported no association between respondents with a high risk of HA and awareness of all five HASs [20]. In the current study, respondents who had risk factors for HA exhibited more awareness of HASs than others did. This result may reflect the effective role of health care professionals in increasing the awareness of HASs in those who had risk factors for CVD.

The findings of this study also demonstrated an association among the following: those who had a family history of HA among their relatives and neighbors; those who received information about HA through public service announcements, social media, the internet, and other sources to become aware that HA requires quick treatment; and all five HASs. This finding is consistent with those of studies conducted in the US and Korea [19,20,25]. Furthermore, in the present study, there were significant differences between those who recognized all five HASs and those who were aware of the appropriate actions to take.

Meanwhile, this research showed no association between age and awareness of all five HASs. These findings, however, are inconsistent with those of studies conducted in Korea and the US that reported differences between younger and older people [19,20,25]. Though the respondents in this study were aged between 18-64 years, most of the respondents were 18-25 years old. Generally, this age group (18-25) is always in good health. Thus, they care less about risk factors for HA and usually have fewer HAs than elderly people do.

### 4.1. Limitations

Convenience sampling and survey bias and interviewer administered survey are a few of the limitations that need to be considered. Moreover, the current findings are from one city in Malaysia and thus cannot be generalized to the whole of the Malaysian population.

### 4.2. Recommendation

It is recommended to have different health programs for individuals who are at high risk of CHD. Mass media campaigns and educational interventions should be aimed at raising awareness of the signs and symptoms of HA, which could familiarize individuals with HA symptoms and risk factors. An educational intervention to directly raise the awareness of people in the high-risk group by means of regular counselling in primary, secondary, and tertiary health care centers as well as advertisement through social media (Facebook, email, YouTube), health messages through cell phones, and health awareness functions on special occasions are some of the recommended future strategies. Health awareness programs on TV; health education campaigns; and meetings in schools, colleges, and universities would also indirectly benefit people at high risk of CVD.

## 5. Conclusions

In this study, the awareness of/and action towards the signs and symptoms of HA were poor among the lay public in Kuantan. Similarly, the majority of the respondents were not aware of all five HAS, whereas the majority of the respondents were aware of chest pain as a HAS while not being aware of other symptoms of HA, and about one third of respondents did not know any HAS. However, more than half of respondents answered “yes” when asked if the trap question is a symptom of HA, which means they did not have knowledge about HAS. One-third of individuals identified calling an ambulance as a suitable action when someone had HAS while only a few respondents identified all five HAS and engaged in appropriate action, calling an ambulance, when someone suffers from HAS.

Improving the awareness and action towards symptoms and signs of HA are urgently required to avoid increased mortality and morbidity. Social media, schools, and medical camp milieus should be considered as valuable sources of information on HA. As such, they should be capitalized to communicate with lay public, given that these means may help to reach the youngest and/or the least educated, who seemed to have lower levels of knowledge.

## Figures and Tables

**Table 1:** Demographic characteristics of participants.

Variables	Frequency	%
Gender		
Male	204	51.9
Female	189	48.1
Age (years)		
18–25	122	31
26–35	78	19.8
36–45	65	16.5
46–55	49	12.5
56–64	79	20.1
Marital Status		
Single	174	44.3
Married	211	53.7
Divorced	5	1.3
Widow	3	0.8
Education		
Primary school	40	10.2
High school	205	52.2
Undergraduate	40	10.2
Diploma	70	17.8
Master	25	6.4
PhD	2	0.5
Others	11	2.8
Employment Status		
House wife	27	6.9
Employed	200	50.9
Self-employed	65	16.5
Unemployed	13	3.3
Student	66	16.8
Retired	22	5.6
Race		
Malay	213	54.2
Chinese	119	30.3
Indian	57	14.5
Others	4	1.0
Monthly income		
less 2000 (≈USD 495)	269	68.4
2000–less 4000 (≈USD 495–985)	81	20.6
4000–6000 (≈USD 985–1480)	42	10.7
more 6000 (<USD 1480)	1	0.3
Medical history		
Hypertension	67	17
Diabetes	50	12.7
Dyslipidemia	61	15.5
Heart diseases (heart attack)	15	3.8
Stroke	7	1.8
Other diseases	4	1

**Table 2:** Awareness of each symptom of heart attack and calling an ambulance.

Percentage % of Answer Yes							
Characteristics	Sudden Pain or Discomfort in the Jaw, Neck, or Back	Weakness or Dizziness	Sudden Pain or Discomfort in the Chest	Sudden Disturbance of Vision in One or Both Eyes (Trap Question)	Sudden Pain or Discomfort in the Arms or Shoulders	Sudden Shortness of Breath	Calling an Ambulance 999
Total	26.35	58.15	71.65	35.95	28.2	68.1	35.6
Gender							
Male	28.9	54.9	67.6	33.8	28.9	63.2	36.8
Female	23.8	61.4	75.7	38.1	27.5	73	34.4
*^D^ p-value*	0.25	0.19	0.07	0.37	0.75	0.03 *	0.62
Age							
18–25	18.9	61.5	70.5	33.6	22.1	70.5	33.6
26–35	25.6	56.4	71.8	39.7	29.5	67.9	26.9
36–45	35.4	64.6	80	40	33.8	72.3	35.4
46–55	26.5	59.2	75.5	42.9	32.7	71.4	42.9
56–64	31.6	48.1	63.3	27.8	29.1	58.2	43
^E^*p*-value	0.11	0.28	0.25	0.34	0.43	0.32	0.21
Marital status							
Single	17.2	58.6	69.5	37.9	25.3	69	33.3
Married	33.2	56.9	73	34.1	28.9	66.8	37.4
Divorce	40	60	60	40	80	60	40
Widow	66.7	100	100	33.3	66.7	100	33.3
*^E^ p-value*	0.00 *	0.50	0.55	0.88	0.02 *	0.62	0.86
Race							
Malay	21.1	64.3	80.3	37.1	23.9	76.5	31
Chinese	34.5	47.9	62.2	32.8	31.1	57.1	42.9
Indian	28.1	54.4	59.6	36.8	38.6	59.6	40.4
Others	50	75	50	50	25	50	0.0
^E^*p*-value	0.04 *	0.02 *	0.00 *	0.80	0.14	0.00 *	0.05
**Education**							
Lower school	25	47.5	57.5	32.5	27.5	55	45
Second school	23.9	57.6	69.3	35.6	28.3	67.3	32.7
Undergraduate	35	70	87.5	40	27.5	82.5	40
Diploma	25.7	60	70	35.7	28.6	65.7	34.3
Master	48	60	88	44	36	76	40
PhD	0.0	50	100	0.0	50	100	0.0
Others	9.1	45.5	72.7	27.3	9.1	63.6	45.5
^E^*p*-value	0.10	0.53	0.03 *	0.84	0.78	0.17	0.61
Monthly income							
Less than 2000	23.8	57.6	70.6	36.8	26.8	66.5	34.9
2000–3999	27.2	60.5	72.8	24.7	30.9	72.8	34.6
4000–6000	40.5	57.1	73.8	52.4	33.3	69	42.9
More than 6000	100	0.0	100	0.0	0.0	0.0	0.0
^E^*p*-value	0.04 *	0.65	0.87	0.01 *	0.68	0.35	0.65
Employment							
Housewife	33.3	51.9	77.8	51.9	22.2	63	44.4
Employed	23.5	57	68.5	39.5	26	67	32
Self-employment	33.8	60	75.4	30.8	41.5	69.2	41.5
Unemployed	23.1	53.8	69.2	15.4	30.8	53.8	46.2
Student	25.8	66.7	74.2	31.8	27.3	74.2	31.8
Retired	27.3	45.5	72.7	22.7	18.2	68.2	45.5
^E^*p*-value	0.62	0.52	0.82	0.09	0.16	0.72	0.41
Hypertension							
Yes	32.8	56.7	67.2	26.9	34.3	68.7	29.9
No	25.2	58.3	72.4	37.7	27	67.8	36.8
^D^*p*-value	0.19	0.81	0.38	0.09	0.22	0.89	0.27
Diabetes							
Yes	40	56	60	26	36	62	42
No	24.5	58.3	73.2	37.3	27.1	68.8	34.7
^D^*p*-value	0.20	0.75	0.05	0.11	0.19	0.33	0.31

* *p*-value <0.05. ^D^ Mann–Whitney. ^E^ Kruskal–Wallis.

**Table 3:** Awareness of heart attack symptoms by the number of the symptoms.

Percentage % of Answer Yes								
Characteristics	≥1	≥2	≥3	≥4	^A^ Five HAS	^B^ Five HAS&C	^C^ 6	Excellent Awareness
Total	81.9	74.3	58.5	26	11.5	5.6	2.8	1.3
Gender								
Male	78.9	71.6	56.9	24.5	11.8	6.4	2.5	1.5
Female	85.2	77.2	60.3	27.5	11.1	4.8	3.2	1.1
^D^*p*-value	0.10	0.19	0.48	0.49	0.83	0.48	0.66	0.71
Age								
18–25	82	73.8	58.2	21.3	8.2	4.1	1.6	0.0
26–35	80.8	74.4	60.3	24.4	11.5	3.8	1.3	0.0
36–45	86.2	81.5	69.2	35.4	13.8	6.2	6.2	4.6
46–55	87.8	79.6	59.2	24.5	14.3	6.1	2	0.0
56–64	75.9	65.8	48.1	27.8	12.7	8.9	3.8	2.5
^E^*p*-value	0.42	0.24	0.15	0.32	0.70	0.62	0.36	0.04 *
Marital status								
Single	81	73.6	58.6	19.5	6.9	3.4	1.1	0.0
Married	82	73.9	57.8	30.3	14.7	7.6	3.8	2.4
Divorce	100	100	60	20	20	0.0	0.0	0.0
Widow	100	100	100	100	33.3	0.0	33.3	0.0
^E^*p*-value	0.60	0.41	0.53	0.00 *	0.05	0.31	0.00 *	0.22
Race								
Malay	89.2	81.2	63.4	24.9	7.5	4.2	1.9	0.9
Chinese	72.3	65.5	50.4	26.9	17.6	8.4	4.2	2.5
Indian	75.4	68.4	57.9	26.3	12.3	5.3	1.8	0.0
Others	75	50	50	50	25	0.0	25	0.0
^E^*p*-value	0.00 *	0.00 *	0.14	0.70	0.03 *	0.42	0.02 *	0.48
Education								
Undergraduate	95	85	67.5	42.5	12.5	10	7.5	5
Diploma	80	74.3	62.9	22.9	10	1.4	2.9	0.0
Master	88	84	64	40	32	16	8	4
PhD	100	100	50	50	0.0	0.0	0.0	0.0
Others	72.7	72.7	45.5	9.1	0.0	0.0	0.0	0.0
^E^*p*-value	0.11	0.37	0.41	0.06	0.04 *	0.11	0.00 *	0.02 *
Monthly income								
Less than 2000	82.2	74	58	22.3	8.9	4.8	1.9	0.4
2000-3999	80	76.5	60.5	33.3	13.6	4.9	1.2	1.2
4000-6000	83.3	71.4	59.5	35.7	23.8	11.9	11.9	7
More than 6000	100	100	0.0	0.0	0.0	0.0	0.0	0.0
^E^*p*-value	0.93	0.86	0.66	0.08	0.03 *	0.31	0.00 *	0.00 *
Employment								
Housewife	88.9	81.5	51.9	18.5	7.4	3.7	3.7	0.0
Employed	79.5	72	56	24.5	10	3.5	2.5	1
Self-employment	84.6	76.9	64.6	36.9	16.9	9.2	3.1	3.1
Unemployed	84.6	61.5	61.5	23.1	0.0	0.0	0.0	0.0
Student	84.8	80.3	66.7	24.2	12.1	6.1	3	0.0
Retired	77.3	68.2	45.5	22.7	18.2	18.2	4.5	4.5
^E^*p*-value	0.73	0.50	0.36	0.37	0.36	0.05	0.97	0.41
Hypertension								
Yes	85.1	73.1	58.2	26.9	16.4	6	3	1.5
No	81.3	74.5	58.6	25.8	10.4	5.5	2.8	1.2
^D^*p*-value	0.46	0.81	0.95	0.85	0.16	0.88	0.91	0.86
Diabetes								
Yes	82	62	54	34	22	10	6	2
No	81.9	76.1	59.2	24.8	9.9	5	2.3	1.2
^D^*p*-value	0.99	0.03 *	0.48	0.16	0.01 *	0.14	0.14	0.62
Dyslipidemia								
Yes	85.2	77	63.9	32.8	21.3	14.8	8.2	4.9
No	81.3	73.8	57.5	24.7	9.6	3.9	1.8	0.6
^D^*p*-value	0.46	0.59	0.35	0.18	0.00 *	0.00 *	0.00 *	0.00 *
History of HA among relatives, acquaintances, or neighbors								
Yes	89	81.3	66.2	31.1	14.6	8.2	4.1	2.3
No	73	65.5	48.9	19.5	7.5	2.3	1.1	0.0
^D^*p*-value	0.00 *	0.00 *	0.00 *	0.01 *	0.02 *	0.01 *	0.07	0.04 *
Heard about heart attack								
Yes	85.9	77.8	61.7	27.8	10.8	5.7	2.7	1.5
No	59.3	54.2	40.7	15.3	15.3	5.1	3.4	0.0
^D^*p*-value	0.00 *	0.00 *	0.00 *	0.04 *	0.32	0.85	0.76	0.34
Received any information related to heart attack by public service announcements and internet								
Yes	84.8	77.2	62.8	31	14.5	7.2	3.8	1.7
No	73.8	66	46.6	11.7	2.9	1	0.0	0.0
^D^*p*-value	0.01 *	0.25	0.00 *	0.00 *	0.00 *	0.01 *	0.04 *	0.18
Being aware heart attack requires prompt treatment								
Yes	86	78.3	62.4	27	11.4	6.3	2.6	1.4
Sometimes	43.5	39.1	17.4	0.0	0.0	0.0	0.0	0.0
No	52.6	42.1	36.8	26.3	26.3	0.0	10.5	0.0
^E^*p*-value	0.00 *	0.00 *	0.00 *	0.00 *	0.02 *	0.24	0.08	0.73

* *p*-value <0.05. ^D^ Mann–Whitney. ^E^ Kruskal–Wallis; HAS = heart attack symptom. ^A^ Aware of five heart attack symptoms. ^B^ Aware of five heart attack symptoms and appropriate action. ^C^ Aware of five heat attack symptoms and trap question response No. Excellent awareness: Aware of heart attack and trap question with response NO and appropriate action “calling ambulance”.(≥1) participant who recognized one HAS. (≥2) those who recognized two HAS. (≥3) participants recognized three HAS. (≥4) participants recognized four HAS.

**Table 4:** Multivariable logistic regression analysis factors related to knowledge of five heart attack symptoms.

Factors	*P*-Value	Odds Ratio	95% Cl
Marital Status			
Single	0.039	0.459	0.219–0.962
Married	0.164	0.117	0.006–2.393
Divorce	0.433	0.221	0.005–9.574
Widowed			
Race			
Malay Non-Malay	0.004	0.381	0.196–0.741
Dyslipidemia			
Yes	0.141	1.865	0.814–4.272
No			
Hypertension			
Yes	0.897	0.940	0.366–2.416
No			
Diabetes			
Yes	0.339	1.604	0.609–4.221
No			
History of HA among relatives, acquaintances or neighbors			
Yes	0.032	4.022	1.126–14.363
No			
Received any information related to HA by public service announcements and or internet			
Yes	0.004	6.095	1.787–20.792
No			
Being aware that HA requires prompt treatment			
Yes	0.280	0.560	0.196–1.604
No			

* Significant with *P* < 0.05.

## Abbreviations

CHD	Coronary Heart Disease
CVD	Cardiovascular Diseases
HA	Heart Attack
IHD	Ischemic Heart Disease
MI	Myocardial Infarction
HAS	Heart attack Symptom
HASs	Heart Attack Symptoms
OR	Odds Ratio
CL	Confidence Interval
KW	Kruskal-Wallis Test
